# iSulf-Cys: Prediction of S-sulfenylation Sites in Proteins with Physicochemical Properties of Amino Acids

**DOI:** 10.1371/journal.pone.0154237

**Published:** 2016-04-22

**Authors:** Yan Xu, Jun Ding, Ling-Yun Wu

**Affiliations:** 1 Department of Information and Computer Science, University of Science and Technology Beijing, Beijing 100083, China; 2 Institute of Applied Mathematics, Academy of Mathematics and Systems Science, Chinese Academy of Sciences, Beijing 100190, China; Harbin Institute of Technology Shenzhen Graduate School, CHINA

## Abstract

Cysteine S-sulfenylation is an important post-translational modification (PTM) in proteins, and provides redox regulation of protein functions. Bioinformatics and structural analyses indicated that S-sulfenylation could impact many biological and functional categories and had distinct structural features. However, major limitations for identifying cysteine S-sulfenylation were expensive and low-throughout. In view of this situation, the establishment of a useful computational method and the development of an efficient predictor are highly desired. In this study, a predictor iSulf-Cys which incorporated 14 kinds of physicochemical properties of amino acids was proposed. With the 10-fold cross-validation, the value of area under the curve (AUC) was 0.7155 ± 0.0085, MCC 0.3122 ± 0.0144 on the training dataset for 20 times. iSulf-Cys also showed satisfying performance in the independent testing dataset with AUC 0.7343 and MCC 0.3315. Features which were constructed from physicochemical properties and position were carefully analyzed. Meanwhile, a user-friendly web-server for iSulf-Cys is accessible at http://app.aporc.org/iSulf-Cys/.

## Introduction

Post-translational modifications (PTMs) play crucial roles in various cell functions and biological processes, as well as in regulating cellular plasticity and dynamics. Cysteine S-sulfenylation in proteins, a reversible covalent oxidation, is one of the posttranslational modifications and has emerged as a dynamic mechanism for inactivation in protein family. It was discovered that the reversible S-sulfenylation modification was involved in various biological processing including cell signaling, response to stress, protein functions and signal transduction.

Identifying S-sulfenylation modification with chemoproteomic approaches [1–4] have been developed and did not give specific modification sites. Meanwhile increasing evidences have demonstrated that the site-specific mapping platform could find broad applications in chemical biology [5]. Yang [6] got over 1000 S-sulfenylation sites on more than 700 proteins through site-specific mapping. However, experimental identification of S-sulfenylation sites with a site-directed mutagenesis strategy is expensive. With the existing experimental data, it is highly desired to develop computational method for timely and reliably identifying the potential S-sulfenylation sites in proteins.

**Fig 1:**
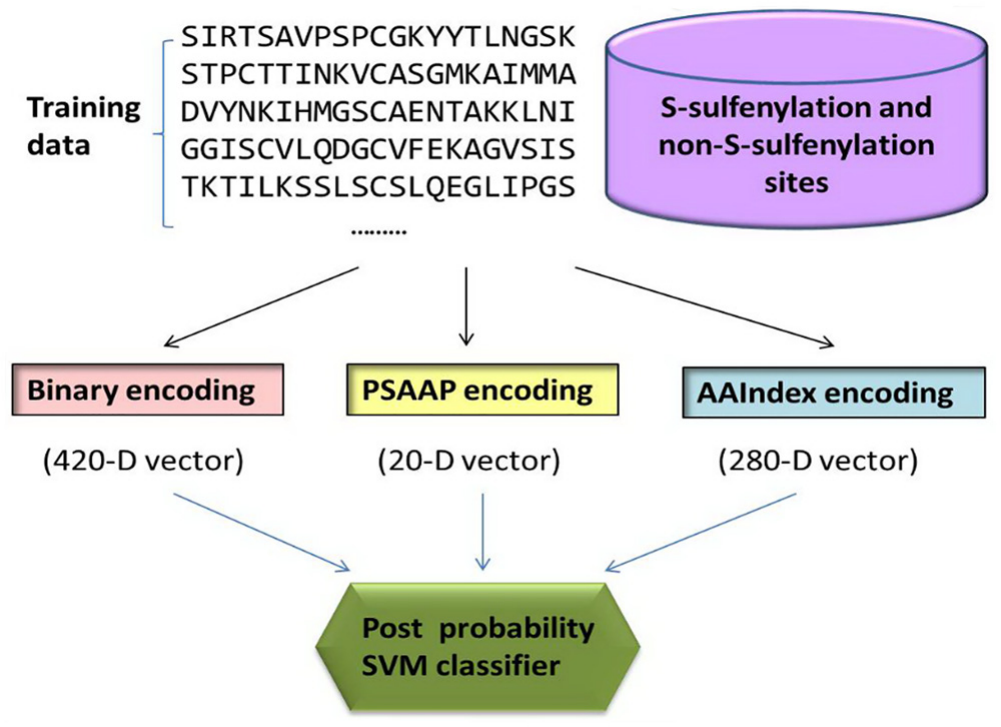
A diagram flow to illustrate the predicting procedure.

The present study was initiated in an attempt to develop a more powerful method to identify the S-sulfenylation sites in proteins. To get the predictor, three different features were constructed from site-specific amino acid propensity, physicochemical and biologic properties. Meanwhile, a user-friendly web-server for the predictor was developed in JAVA. We hope that the online web-sever could become a useful tool for both basic research and drug development in the relevant areas. Fig 1 is the chart to illustrate the prediction procedure.

## Materials and Methods

### Data collection and preprocessing

To develop a statistical predictor, it is fundamentally important to establish a reliable and rigorous benchmark dataset to train and test the predictor. The benchmark dataset which contains some errors will lead to an unreliable predictor and the accuracy tested could be completely meaningless. The experimentally validated S-sulfenylation cysteine benchmark dataset used in this study was derived from[6]. A total of 1105 S-sulfenylated sites on 778 Homo proteins identified in RKO cells from quantitative S-sulfenylome analyses. Only the canonical protein isoforms are retained. The corresponding protein sequences were retrieved from NCBI database. To facilitate description later, for every peptide fragment **P** with cysteine (C) located at its center, it can be expressed as

$$P={\text{R}}_{-\xi }{\text{R}}_{-(\xi -1)}\cdots {\text{R}}_{-2}{\text{R}}_{-1}\text{C}{\text{R}}_{1}{\text{R}}_{2}\cdots {\text{R}}_{\left(\eta -1\right)}{\text{R}}_{\eta }$$ (1)

where the subscript ξ, η are integers, R_−ξ_ represents the ξ-th uptream amino acid residue from the center, R_η_ the η-th downstream amino acid residue, and so forth.

The number of the upstream and downstream amino acid residues has been calculated from the experimental peptides and their average lengths of upstream and downstream are 5.838 ± 4.741 and 6.988 ± 4.514, respectively. So ξ = η = 10 was adopted. If the upstream or downstream in a peptide was less than 10, the lacking residues were filled with a dummy residue ‘‘X”. The peptide **P** with an experimentally S-sulfenylated site was defined as positive sample and other peptides with cysteine at center in the same experimental proteins were defined as negative samples.

To reduce the redundancy and avoid homology bias which would overestimate the predictor, we removed those peptides that had ≥ 40% pairwise sequence identity to any other from the benchmark datasets. Finally, we obtained the benchmark dataset which contained 1045 S-sulfenylated and 7124 non-S-sulfenylated peptide samples.

To further demonstrate and verify the performance of the predictor, we randomly divided the dataset into two subsets S_tr and S_te which were used for training and testing, respectively. Training dataset S_tr contained 900 S-sulfenylated peptides and 6856 non-S-sulfenylated peptides which were randomly derived from dataset, respectively. The independent testing dataset S_te contained the remaining 145 S-sulfenylated peptides and 268 non-S-sulfenylated peptides which none of them was in the training dataset S_tr. The description of the dataset was in Table 1. All the experimental S-sulfenylation peptides and their modified sites were listed in S1 Data.

**Table 1:** The number of positive and negative peptides in training and independent test dataset.

*Data*	*Positive*	*Negative*
S_tr	900	6856
S_te	145	268

### Feature Construction

In the theme of using machine learning methods to predict posttranslational modification sites (PTMs), the feature construction was an important processing which would depend on how to extract the desired information from the peptide sequences. Amino acid physicochemical properties and position-specific amino acid propensity were utilized to convert peptide fragments into feature constructions. As the center position in peptides was always cysteine (C), we omitted it in the encoding schemes. In fact there were 20 amino acid residues participating in feature construction in a peptide.

#### (a)Binary encoding

Binary feature construction is the orthogonal binary encoding scheme which translates every amino acid into a 20-dimensional vector. For example, alanie (A) was encoded as “10000000000000000000”, cysteine (C) was “01000000000000000000” and so on. There were 21 amino acid residues (20 native and 1 pseudo ‘X’) in our dataset. The alanie (A) was encoded as “100000000000000000000”(a 21 dimensional vector), cysteine (C) was “010000000000000000000”,…, X was “000000000000000000001”. We got a 20*21 = 420 dimensional vector for a peptide **P**.

#### (b)The position-specific amino acid propensity

The position-specific amino acid propensity (PSAAP) has been introduced in [7] which used 20 native amino acids and got excellent results. The PSAAP matrix was 21*20 which every row denoted one kind of amino acids and the column denoted positions in a peptide. We used this encoding scheme and got a 20 dimensional vector for every peptide **P**.

#### (c) AAIndex property

Each amino acid has many specific physicochemical and biologic properties. These properties have direct or indirect effects on protein properties. Different combinations of those properties have different influences to the structures and functions of proteins. AAIndex [8] is a database which contains various physicochemical and biologic properties of amino acids. Some combinations of physicochemical properties have been utilized which transformed sequence fragments into mathematical vectors and have shown efficient effects [9, 10]. In this work, we selected fourteen physicochemical properties from AAIndex database, including hydrophobicity, solvent, polarity, polarizability, accessible, *PK-N*, *PK-C*, melting point, molecular weight, optical rotation, net charge index of side chains, entropy of formation, heat capacity and absolute entropy. The pseudo amino acid X was defined 0 as its physicochemical property value. Therefore, each amino acid was constructed into 14 features through AAIndex database. For a peptide fragment, a 280-D (20*14 = 280) feature vector was obtained through AAIndex encoding scheme. The number of the three different feature constructions was given in Table 2.

**Table 2:** The number of dimensions of three feature constructions.

*Features*	*AAIndex*	*Binary*	*PSAAP*
*No*.	280	420	20

### Algorithm

For the prediction of cysteine S-sulfenylation sites in proteins, the support vector machine (SVM) algorithm was used and the post probability SVM was implemented by LIBSVM[11], a public and widely used SVM library. In this work, the kernel function was radial basis function (RBF) kernel with parameter g = 0.005. For a query peptide **P** as formulated by feature construction, suppose *p*_*r*_ is its probability to the S-sulfenylated peptide. The query peptide **P** is predicted as a S-sulfenylation modification if *p*_*r*_ is greater than a cutoff, otherwise non-S-sulfenylation. The cutoff value is default 0.5 for balancing the true positive and negative rate. The predictor established via the above procedures was called iSulf-Cys.

### Five metrics for measuring prediction quality

To illustrate the performance of the statistical predictor, we utilized the four common measurements. The four frequent measurements are sensitivity (SN), specificity (SP), accuracy (ACC), and Mathew correlation coefficient (MCC). They are defined as

$$\{\begin{array}{l} \text{SN}=\frac{\text{TP}}{\text{TP+FN}}\\ \text{SP}=\frac{\text{TN}}{\text{TN+FP}}\\ \text{ACC}=\frac{\text{TP+TN}}{\text{TP+TN+FP+FN}}\\ \text{MCC}=\frac{\left(\text{TP}\times \text{TN})-(\text{FP}\times \text{FN}\right)}{\sqrt{\text{(TP+FP)(TP+FN)(TN+FP)(TN+FN)}}} \end{array}$$ (2)

where TP (true positive) represents the number of S-sulfenylated peptides correctly predicted, TN (true negative) the numbers non-S-sulfenylated peptides correctly predicted, FP (false positive) the non-S-sulfenylated incorrectly predicted as the S-sulfenylated peptides, and FN (false negative) the S-sulfenylated peptides incorrectly predicted as the non-S-sulfenylated peptides. In addition to the above four criteria, the AUC (area under the receiver operating characteristic curve) is also utilized as a quantitative indicator of robustness.

## Results and Discussion

### The evaluation of the prediction performance and accuracy

In statistical prediction, the following three cross-validation methods are often used to examine a predictor for its performance in practical application: independent test, subsampling or K-fold (such as 6-fold, 8-fold, or 10-fold) cross-validation test and the leave-one-out (LOO) cross-validation. The LOO always yielded a unique result for a given benchmark dataset and has been widely used in PTM sites [12–16] and various statistical predictors [17–19] because it was the most unbiased. The K-fold cross-validation for its shorter computational time has also been utilized in literatures [20–22]. In this work 10-fold cross-validation has been adopted and was performed 20 times for different subsampling combinations, followed by averaging their outcomes. The last results were mean ± standard variance.

**Fig 2:**
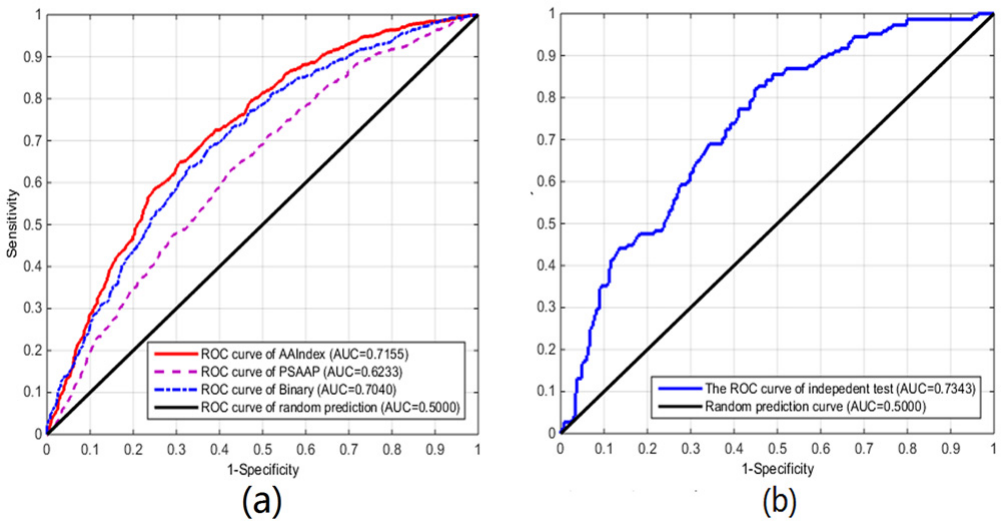
(a)The 10-fold ROC curves of the three feature constructions on the balanced training dataset. (b) The 10-fold ROC curve of AAIndex feature construction on the independent test.

The results which were obtained on the training dataset were given in Table 3 with the four metrics as defined in Eq 2. The Table 3 also contained the results of three different feature constructions. As can be seen from Table 3 and Fig 2(a), the overall AUC was 0.7155 ± 0.0085 for the AAIndex which were higher than PSAAP (0.6233 ± 0.0054) and Binary (0.7040 ± 0.0083) encoding schemes. Meanwhile the accuracy, sensitivity, specificity and MCC for AAIndex were (65.59 ± 0.72)%, (67.31 ± 0.73)%, (63.89 ± 1.05)% and 0.3122 ± 0.0144 on training dataset. MDD-SOH[23] is an another existing S-sulfenylation predictor based on the same data[6]. The results were listed in Table 3 in 5-fold cross-validation which the training data were 1031 positive and 216 negative samples. The two predictors have the comparable performances on the S-sulfenylation sites.

**Table 3:** The 10-fold cross-validation results of three different feature constructions on the balanced training dataset. The results have been run 20 times for every feature construction by SVM algorithm with g = 0.005 and cutoff = 0.5. The values are mean ± standard variance. The results of MDD-SOH were obtained in 5-fold cross-validation.

*Features*	*AUC*	*SN*(*%*)	*SP*(*%*)	*ACC*(*%*)	*MCC*
PSAAP	0.6233 ± 0.0054	31.34 ± 1.52	81.74 ± 0.75	56.54 ± 0.55	0.1515 ± 0.0114
Binary	0.7040 ± 0.0083	68.56 ± 0.47	63.11 ± 0.87	65.83 ± 0.67	0.3172 ± 0.0135
AAIndex	**0.7155** ± **0.0085**	67.31 ± 0.73	63.89 ± 1.05	65.59 ± 0.72	0.3122 ± 0.0144
MDD-SOH	--	68	70	70	0.27

On the independent test which none of them was in the training dataset, the AUC was 0.7343 and MCC 0.3315 (see Table 4 and Fig 2(b)). Fig 2 showed the performance of the proposed predictor.

**Table 4:** The 10-fold cross-validation results of independent test by SVM algorithm with g = 0.005 and cutoff = 0.5.

*AUC*	*SN*(*%*)	*SP*(*%*)	*ACC*(*%*)	*MCC*
0.7343	68.97	65.67	66.83	0.3315

### The feature construction analysis for amino acids

**Fig 3:**
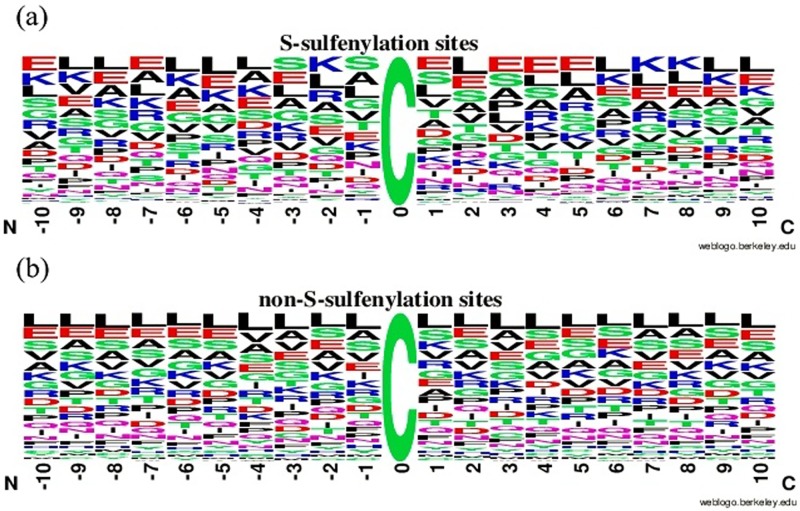
(a) The amino acid composition Logo of S-sulfenylated peptides. (b) The amino acid composition Logo of non-S-sulfenylated peptides.

**Fig 4:**
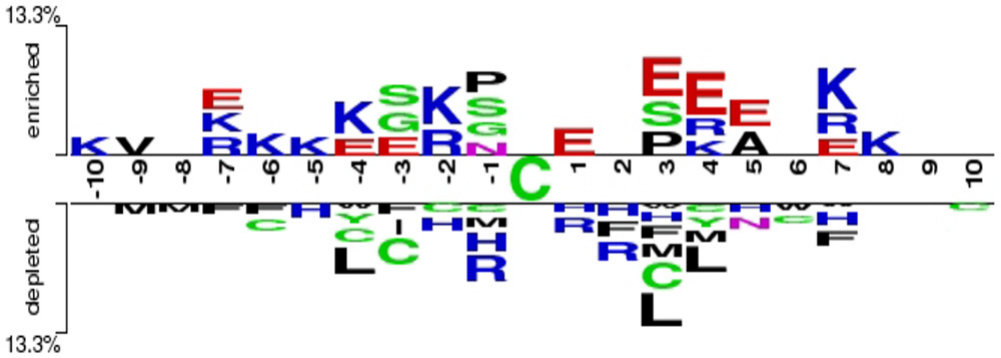
The TwoSampleLogo between sulfenylation and non-sulfenylation peptides (p<0.01).

Amino acid composition was utilized to illustrate differences between S-sulfenylation and non-S-sulfenylation peptides. The WebLogo [24] (Fig 3) clarified the amino acid compositions for the peptides which could not obviously demonstrated the differences between S-sulfenylated and non-S-sulfenylated peptides. Another clear and succinct TwoSampleLogo [25] (Fig 4) revealed the differences from statistically significant differences (*p*<0.01). It showed that the lysine (K), arginine (R), glutamic (E) in the upstream and lysine (K), glutamic (E) in the downstream played an important role in S-sulfenylated peptides. While the leucine (L) residue played a relative role in the non-S-sulfenylated peptides. The lysine (K) (at position -6, -5,-4,-2,+7 and +8) and arginine (R) (at position -2, -4) are positive polar residues and glutamic (E) (at position -4,-3,+1,+3,+4 and +5) is negative polar residue in the S-sulfenylated peptides. Meanwhile leucine (L) is nonpolar residue in the non-S-sulfenylated peptides at the position -4 and +3. All these indicated that the position-specific propensities and physicochemical properties played intrinsic effects in the discriminant between S-sulfenylated and non-S-sulfenylated peptides.

### The online web-service of iSulf-Cys

**Fig 5:**
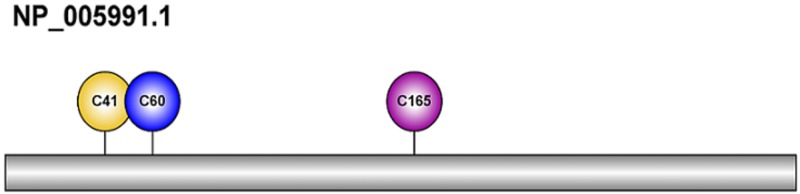
The predictive IBS results of the online webserver.

A user-friendly and publicly accessible web-server is one of the keys in the statistical prediction of posttranslational modification. For the convenience of the vast majority of experimental scientists, we have developed a web-server for the iSulf-Cys predictor in JAVA. Users can easily get their desired results from the online webserver. The input proteins should be in FASTA format and the output with IBS[26] software as Fig 5. The web-server can be freely accessible at http://app.aporc.org/iSulf-Cys/.

## Discussion and Conclusions

One particular challenge in machine learning such as support vector machine and conditional random forest is that the available dataset was highly unbalanced: the number of S-sulfenylation peptides (positive instances) is much smaller than the number of non-S-sulfenylation peptides (negative instances). Unbalanced dataset presents a challenge for support vector machine classifier that is trained to optimize the generalization accuracy. Standard support vector machine algorithm without considering class-imbalance leads to high false negative rate by predicting the positive as the negative one [27, 28]. In order to overcome this disadvantage, a common approach is to change the distribution of positive and negative instances during training by randomly selecting a subset of the training data from the majority class. Following the approach used in the literatures [29, 30], we balanced the positive and negative dataset during the cross-validation by randomly selecting the negative sequence peptides from the whole negative dataset for 20 times.

As one of the new posttranslational modifications (PTMs) for cysteine (C), S-sulfenylation could impact many biological and functional categories. The predictor iSulf-Cys was developed for identifying the cysteine S-sulfenylation in proteins. The benchmark dataset was entirely derived from site-specific mapping experiments. Forteen physicochemical properties were took into account in feature constructions which polar attribute displayed strong power between S-sulfenylation and non-S-sulfenylation. The proposed predictor also showed good performance in independent test. Meanwhile an online web-server http://app.aporc.org/iSulf-Cys/ was developed for the predictor which would facilitate the use for the biologists.

## Supporting Information

S1 Data The dataset contained non-homologous 1045 S-sulfenylated and 7124 non-S-sulfenylated cysteine peptides which had been retrieved from 778 Homo proteins. (XLSX)
